# Chronic Hypertension in Pregnancy and Placenta-Mediated Complications Regardless of Preeclampsia

**DOI:** 10.3390/jcm13041111

**Published:** 2024-02-16

**Authors:** Yair Cohen, Gil Gutvirtz, Taeer Avnon, Eyal Sheiner

**Affiliations:** 1 Department of Internal Medicine, Soroka University Medical Center, Beer-Sheva 84101, Israel; yaircoh@clalit.org.il; 2 Department of Obstetrics and Gynecology, Soroka University Medical Center, Beer-Sheva 84101, Israel; gilgut@bgu.ac.il (G.G.); avnont@bgu.ac.il (T.A.); 3 Faculty of Health Sciences, Ben-Gurion University of the Negev, Beer-Sheva 84105, Israel

**Keywords:** chronic hypertension, pregnancy, placenta, complications, maternal, fetal

## Abstract

Background: The prevalence of chronic hypertension in women of reproductive age is on the rise mainly due to delayed childbearing. Maternal chronic hypertension, prevailing prior to conception or manifesting within the early gestational period, poses a substantial risk for the development of preeclampsia with adverse maternal and fetal outcomes, specifically as a result of placental dysfunction. We aimed to investigate whether chronic hypertension is associated with placenta-mediated complications regardless of the development of preeclampsia in pregnancy. Methods: This was a population-based, retrospective cohort study from ‘Soroka’ university medical center (SUMC) in Israel, of women who gave birth between 1991 and 2021, comparing placenta-mediated complications (including fetal growth restriction (FGR), placental abruption, preterm delivery, and perinatal mortality) in women with and without chronic hypertension. Generalized estimating equation (GEE) models were used for each outcome to control for possible confounding factors. Results: A total of 356,356 deliveries met the study’s inclusion criteria. Of them, 3949 (1.1%) deliveries were of mothers with chronic hypertension. Women with chronic hypertension had significantly higher rates of all placenta-mediated complications investigated in this study. The GEE models adjusting for preeclampsia and other confounding factors affirmed that chronic hypertension is independently associated with all the studied placental complications except placental abruption. Conclusions: Chronic hypertension in pregnancy is associated with placenta-mediated complications, regardless of preeclampsia. Therefore, early diagnosis of chronic hypertension is warranted in order to provide adequate pregnancy follow-up and close monitoring for placental complications, especially in an era of advanced maternal age.

## 1. Introduction

Hypertension is a chronic, age-dependent disease most common in older populations. Due to its chronic course and long-term effects, the disease has a heavy medical burden. It is a significant risk factor for multi-system injury, including damage to the central and peripheral nervous systems, cardiovascular, renal, and endocrine systems [1,2]. The most common injuries resulting from chronic hypertension are nervous and cardiovascular system damage [2] and are due to an increased risk of atherosclerosis and thrombophilia [3]. Despite the chronic, long-standing nature of the disease, hypertension does not only affect the elderly population but can also affect the younger population with relatively shorter disease duration, and it has a pivotal impact on pregnant women. 

Hypertension may result in continuous injury to endothelial cells and small blood vessels required for placental development during early pregnancy and places the pregnancy at an increased risk of developing placenta-mediated pregnancy complications. The process of placental development and implantation relies on the development of a highly delicate, branching vascular network of the spiral arteries (found at the depth of the uterine myometrium) to enable vasodilation and increased placental perfusion. When this process is interrupted due to pre-existing vascular damage, the intricate vascular alterations induced by chronic hypertension contribute to compromised placental perfusion, culminating in aberrant placental function and structure that may lead to placenta-mediated pregnancy complications, notably preeclampsia, placental abruption, and impaired fetal growth [4].

Chronic hypertension complicates 1% to 2% of pregnancies and constitutes one of the highest risk factors among maternal characteristics and medical history for the development of preeclampsia [5,6]. Maternal chronic hypertension has also been suggested to enhance the risk for other pregnancy complications such as gestational diabetes, fetal growth restriction (FGR), and increased fetal and maternal morbidity and mortality [7,8,9]. For example, a 2014 systematic review and meta-analysis by Bramham et al. found that women with chronic hypertension had a substantially increased risk (compared with women from the US national population dataset) for preterm delivery, cesarean birth, low birthweight (<2500 g) infants, and an increased risk for perinatal mortality [10].

In a large cohort study of nearly 2.8 million singleton deliveries in Sweden, Khalaf et al. [11] investigated pregnancy outcomes of women with chronic kidney disease, chronic hypertension, or both conditions. In their large cohort of deliveries, they found that chronic hypertension was associated with a higher risk for cesarean delivery, a small risk for gestational age (SGA) infant, and an increased risk for stillbirth, but the strongest association for chronic hypertension was with the development of preeclampsia in pregnancy (superimposed preeclampsia) [11]. Superimposed preeclampsia (a new development of symptoms suggestive of preeclampsia in women with preexisting, chronic hypertension) occurs in about 20% of women with chronic hypertension and in superimposed preeclampsia, compared with preeclampsia alone, there is a higher incidence of adverse maternal and perinatal outcomes [12,13,14]. However, not all studies provide definitive conclusions, and some are based on relatively small numbers [15].

In addition to the obstetrical and perinatal complications described above, chronic hypertension in pregnant women acts as a significant risk factor for long-term complications to both the mother and newborn. For the mother, women with chronic hypertension were found to be at an increased risk for developing cardiovascular disease with increased mortality [16]. The newborn is at increased risk of significant long-term disease later in life, such as cardiovascular disease and metabolic disease, including congenital heart disease and childhood obesity [9,17].

In recent years, we have witnessed an increase in the prevalence of primary hypertension among women of reproductive age [18]. This increase is likely due to a rise in the number of women who delay pregnancy to an older age and an increased prevalence of obesity in this population. This trend is expected to continue in the upcoming years [18,19]. 

This study aimed to expose maternal risk factors for chronic hypertension and examine the correlation between chronic hypertension and adverse pregnancy outcomes, specifically placenta-mediated pregnancy complications, regardless of preeclampsia.

## 2. Materials and Methods

This was a retrospective cohort study investigating maternal risk factors for chronic hypertension and comparing pregnancy outcomes, specifically placenta-mediated complications, between parturients with chronic hypertension (exposure) and parturients without chronic hypertension. The diagnosis of maternal chronic hypertension refers to preexisting hypertension (diagnosed before pregnancy) or elevated blood pressure diagnosed during the first 20 weeks of pregnancy. This definition was used for women who had at least two blood pressure measurements that exceeded either 140 mmHg systolic pressure or 90 mmHg diastolic pressure on two different occasions, or had a diagnosis of chronic hypertension listed in their medical file prior to pregnancy. These criteria are in accordance with the American College of Obstetricians and Gynecologists (ACOG) guidelines [20]. We had no access to the participants’ medication list, so this aspect was not investigated. 

Regarding the basic definition of hypertension, although the diagnostic criteria for hypertension were revised during the years of the study in other disciplines (e.g., the updated guidelines of the American College of Cardiology (ACC) and the American Heart Association (AHA)), the obstetrical community acknowledges the newly released recommendations but have not yet redefined their diagnostic criteria. Both the European Society of Cardiology (ESC) and Hypertension Canada, whose task forces also published guidelines for the management of cardiovascular diseases during pregnancy since the AHA/ACC recommendations changed in 2017, have also not changed their diagnostic criteria [21].

Only singleton deliveries were included. Multiple gestations and fetuses with anatomical or chromosomal abnormalities were excluded from the study. A flow chart describing the inclusion and exclusion criteria can be found in the Supplementary Materials Figure S1. The data were taken from a large, single-center database of women who gave birth at Soroka University Medical Center (SUMC) between 1991 and 2021. SUMC is the largest birthing center in Israel, with 17,000 births per year. The hospital is the only tertiary care center in the Negev region and is the main provider of care for the diverse population residing in the area. Therefore, the data were based on a non-selective population. The SUMC perinatal database includes maternal epidemiological and obstetrical information, including demographic details and information on pregnancy course and outcome.

Maternal characteristics included maternal age and parity as continuous variables. Obesity (defined as BMI ≥ 30 kg/m^2^), smoking status (self-report), use of fertility treatments (including ovulation induction protocols and in vitro fertilization (IVF) treatments), and pre-gestational diabetes mellitus were all defined as binary variables (yes/no). 

The placenta-mediated outcomes evaluated in this study were as follows: fetal growth restriction (FGR), defined as estimated fetal weight (EFW) < 10th percentile for gestational age and gender; placental abruption defined by the clinical characteristics of vaginal bleeding with non-reassuring fetal heart rate (NRFHR) pattern and a manual evaluation of the placenta by the attending obstetrician or midwife when feasible; preterm delivery defined as delivery prior to completed 37 weeks’ gestation; preeclampsia defined as at least two BP readings exceeding 140 mmHg systolic and/or 90 mmHg diastolic BP at least 4 h apart with proteinuria (diagnosed via dipstick test + 1 or higher, protein/creatinine ratio ≥ 0.3 or a 24 h urine collection with protein level ≥ 300 mg); and perinatal mortality, including stillbirth (intrauterine fetal demise (IUFD) ≥ completed 22 weeks’ gestation), intrapartum fetal death, or postpartum (up to 30 days post-delivery) infant death. Importantly, for women with chronic hypertension, superimposed preeclampsia was diagnosed based on the new development of thrombocytopenia, liver dysfunction, renal insufficiency, or symptoms suggestive of PE in women with chronic hypertension such as newly uncontrolled hypertension and/or the presence of new-onset proteinuria based on the same criteria elicited above. These criteria are in accordance with ACOG [20] and commonly used in other studies [14]. 

**Figure 1:**
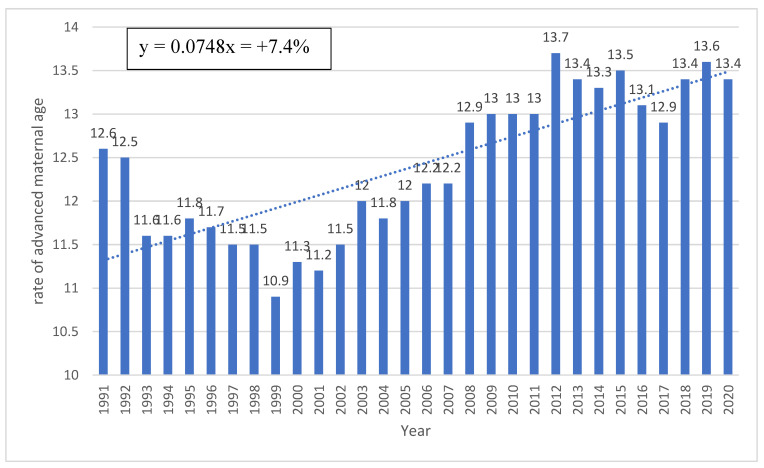
Rates of advanced maternal age (% of study population per year).

**Figure 2:**
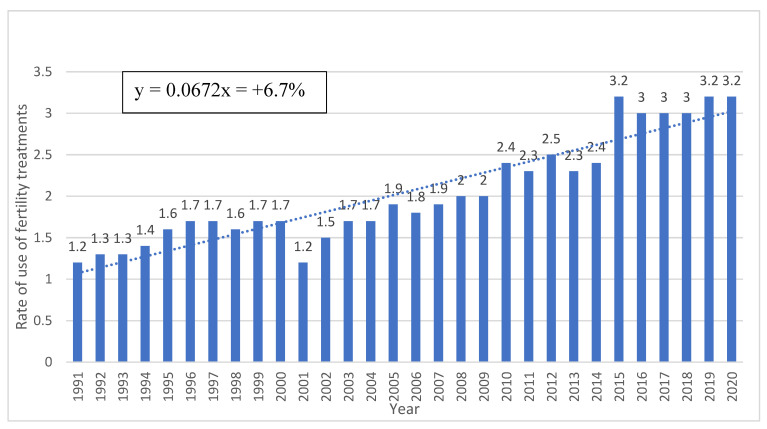
Rates of use of fertility treatments (% of study population per year).

Since this study had a very long follow-up (30 years), we also analyzed several outcomes for trends during the study period. These are shown in Figure 1 and Figure 2 with the calculated mean annual increase/decrease (slope of the curve). Trends over the years of the study of the placenta-mediated complications investigated in the study are also available in the Supplementary Materials (Figures S2–S6).

### Statistical Analysis

Statistical analysis was performed using SPSS package 29th ed. (IBM/SPSS, Chicago, IL, USA). Categorical data (shown as counts and rates, with the differences assessed using chi-square for general associations. Student *t*-test was used for comparison of continuous variables with a normal distribution (maternal age and birthweight). For each of the placenta-mediated outcomes that were found statistically significant in the univariate model, a generalized estimating equation (GEE) model was used to control for siblings (repeated deliveries of mothers) and potential confounders based on the univariate analysis as well as variables of clinical significance, and included the following: maternal age, smoking status, use of fertility treatment, diabetes mellitus (pre-gestational or gestational), obesity, parity, and year of inclusion to the study (child birth year). Using cluster analysis for each parturient and not as a cluster of total births (each parturient = cluster with several births) enables us to eliminate deviation of multiparous women with differing numbers of births and to control for the influence of women who are at an increased or decreased risk for placenta-mediated complications in the remainder of their pregnancies. Including the child birth year in the model also accounts for changes in policies or management that might have occurred during the study period. All analyses were two-sided, and a *p*-value of ≤0.05 was considered statistically significant.

## 3. Results

A total of 356,356 deliveries that occurred in SUMC between 1991 and 2022 met our inclusion criteria. A flow chart describing the inclusion and exclusion criteria can be found in the Supplementary Materials Figure S1. Among them, 3949 (1.1%) deliveries were of mothers who were diagnosed with chronic hypertension. Table 1 presents a summary of the demographic data of women with and without chronic hypertension and reveals some of the risk factors for maternal chronic hypertension in this population. Women with chronic hypertension tended to be older and have comorbidities such as obesity and diabetes mellitus (pre-gestational). They were also more likely to have undergone fertility treatments than their counterparts who were significantly younger and more likely nulliparous.

Table 2 presents pregnancy outcomes for both study groups. Women with chronic hypertension had a lower mean gestational age at birth than women without chronic hypertension. About 40% of pregnancies of women with chronic hypertension were medically induced into labor in comparison with the majority of women without chronic hypertension who went into spontaneous labor. Gestational diabetes mellitus was also more common in the chronic hypertension group. Women with chronic hypertension were more prone to suffer from placenta-mediated complications, including FGR, placental abruption, preeclampsia, and preterm delivery. They were also more likely to have a cesarean delivery, and their offspring had higher rates of low (<7) Apgar scores. Perinatal mortality rates were also significantly higher in women with chronic hypertension. 

Table 3 describes the results of the GEE models that were performed for each placenta-mediated complication investigated in this study. All GEE models were adjusted for preeclampsia, maternal age, smoking status, use of fertility treatment, diabetes mellitus (pre-gestational or gestational), obesity, parity and year of inclusion to the study (child birth year). With the exception of placental abruption, the models reaffirm that chronic hypertension is an independent risk factor for placenta-mediated complication including FGR (adjusted odds ratio (aOR) 1.59, 95%CI 1.40–1.79, *p* < 0.001), preterm delivery (aOR 2.29, 95%CI 2.09–2.52, *p* < 0.001), and perinatal mortality (aOR 1.66, 95%CI 1.25–2.21, *p* < 0.001). In another GEE model adjusted for maternal age, smoking status, use of fertility treatment, diabetes mellitus (pre-gestational or gestational), obesity, parity, and year of inclusion to the study (child birth year), we found that chronic hypertension was also an independent risk factor for the development of superimposed preeclampsia (aOR 3.58, 95%CI 3.18–4.03, *p* < 0.001).

The following figures represent rates of advanced maternal age (>35 years), use of fertility treatments, and rates of chronic hypertension trends during the 30 years of the study period. Figure 1 presents annual rates of advanced maternal age (>35 years) among the group of women with chronic hypertension. During the study period, an average annual increase of 7.4% was noted.

Figure 2 presents annual rates of the use of fertility treatments among the group of women with chronic hypertension. During the study period, an average annual increase of 6.7% was noted.

Additional figures representing the annual trends of the placenta-mediated outcomes investigated in this study can be found in the Supplementary Material (Figures S2–S6).

## 4. Discussion

Our study found a significant association between chronic hypertension in pregnancy and various placenta-mediated complications, even after carefully controlling for known risk factors such as maternal age, smoking, and diabetes mellitus. We found that the rates of preeclampsia, FGR, placental abruption, preterm delivery, and perinatal mortality were all increased in this cohort of women with chronic hypertension, regardless of the presence of superimposed preeclampsia. 

Hypertension is a disease of vascular origin. The hormonal changes of pregnancy require significant adaptations in the cardiovascular physiology of the mother beginning early in the first trimester. In short, surges of estrogen, progesterone, and relaxin leads to systemic vasodilation to encounter the expansion in plasma volume that accompany early pregnancy. Increased heart rate and stroke volume elevate cardiac output, which compensates for the decline in vascular resistance in order to maintain blood pressure at high-enough levels for maternal and placental perfusions [23].

In healthy individuals, balance is gained through adequate vascular response to blood volume changes. However, in women with chronic hypertension and presumed preexisting systemic vascular endothelial dysfunction, this adaptation process is met with vascular resistance that probably leads to a less effective cytotrophoblastic invasion of the uterine spiral arteries, which results in placental hypoxia. It is hypothesized that chronic placental hypoxia is the key factor for placenta-mediated complications [20].

Placenta-mediated complications encountered in affected pregnancies have been evaluated in many studies, systemic reviews, and meta-analyses that investigated the broad spectrum of hypertensive disorders of pregnancy [6,10,11]. In this study, we focused on chronic hypertension, an age-dependent disease, which we believe is becoming increasingly more relevant for the obstetric medical community. As a disease of vascular origin, the effect of chronic hypertension on pregnancy is critical, with negative consequences that can develop in both fetus and mother, with the potential harm being twofold: significant long-term damage to maternal and fetal quality of life and decreased life expectancy [16,17]. Therefore, diagnosing chronic hypertension in young women of reproductive age is crucial. Early diagnosis allows us to label women as high-risk for developing the complications described above and provide prenatal care directed towards the wide range of placenta-mediated complications that influence both mother and fetus. Thus, we can provide the proper and immediate medical response to pregnancy complications that may arise, as well as informed counseling for cardiovascular, metabolic, and endocrine disorders that can develop for years after the end of pregnancy in the mother and newborn, as previously described. 

The prevalence of essential hypertension in women of reproductive age is globally on the rise, which may be attributable to a delay in childbearing to older age and an increased prevalence of obesity in this population [18,19]. On the other hand, hypertension awareness, treatment, and control have improved substantially in high-income countries over the last 3 decades [24]. We found that advanced maternal age, obesity, and the use of fertility treatments were more prevalent in women with chronic hypertension. Therefore, awareness of existing risk factors is important in screening for the disease. The fact that women with chronic hypertension are generally older and have higher rates of obesity is in accordance with previous studies on the subject [1,5], but the higher rates of fertility treatment, with increased use in the recent years, is still considered controversial [25,26]. However, infertility rates correlate with advanced maternal age, so this association can be considered trivial. This trend, which was also demonstrated in our study (Figure 1 and Figure 2), is expected to grow in upcoming years, with the medical burden of these complications on future health aspects expected to rise simultaneously [18,19]. To note, smoking rates were comparable between our study groups and seems extremely low (0.7%). This variable was collected through self-reporting, which might explain the low rates of smoking in both study groups. However, smoking is a well-established risk factor for pregnancy complications and is also associated with placenta-mediated complications, so we decided to include this variable and also adjust for it in the GEE models. 

In accordance with previous studies such as the large cohort study of Khalaf et al. [11] and the systematic review and meta-analysis by Bramham et al. [10], we found that chronic hypertension is an independent risk factor for various placenta-mediated complications including superimposed preeclampsia, FGR, preterm delivery, and perinatal mortality. While the study by Khalaf et al. cited above adjusted for many maternal characteristics (age, parity, etc.) and comorbidities (diabetes mellitus, cardiovascular morbidity, and asthma), they did not account for superimposed preeclampsia itself to be in some part related to the other placenta-mediated complications. As discussed in the introduction section, chronic hypertension is strongly associated with the development of superimposed preeclampsia, which, in turn, constitutes one of the most crucial risk factors for adverse perinatal outcomes. In patients with chronic hypertension, preeclampsia tends to have an earlier onset and to be more severe, and the prognosis for the woman and her fetus is worse than in either condition alone [8,27]. Hence, in our study, we carefully adjusted for superimposed preeclampsia in our models for each of the placenta-mediated complications that we investigated. These models revealed that the risk for placental-related complications is independent and regardless of preeclampsia. 

Importantly, the risk for perinatal mortality which is probably the most devastating outcome in pregnancy was also found to be independently associated with chronic hypertension with an adjusted hazards ratio of 1.66. Our results are in line with the systematic review and meta-analysis by Bramham et al. [10], which also found a 4.2 RR for perinatal death in their cohort. This further highlights the importance of the close monitoring these high-risk women with an even increased surveillance when getting closer to term for planning a safe and timely delivery. 

Our findings stress the importance of implementing practical recommendations in several aspects. The first is to initiate active screening for hypertension in the young population, and, specifically, those with the above-mentioned risk factors, before the beginning of pregnancy, and to emphasize home monitoring of blood pressure, since chronic hypertension is an asymptomatic disease. In accordance with international guidelines, women with chronic hypertension should be evaluated pre-pregnancy to identify possible maternal comorbidities (e.g., obesity, diabetes) before pregnancy. Importantly, those with modifiable risk factors may benefit from counseling on weight loss, diet (reducing excessive sodium and caffeine intake), and lifestyle modifications (smoking cessation) [21].

Second, a patient diagnosed with chronic hypertension in pregnancy should receive early counseling and prenatal care to directly monitor for the described complications. Timely interventions encompassing close maternal surveillance, blood pressure regulation, and fetal assessments are paramount in mitigating the risks associated with chronic hypertension and placenta-mediated complications. Clear guidelines for the use of anti-hypertensive medications to control blood pressure in pregnancy are still lacking due to controversy regarding their efficacy at improving perinatal outcomes; however, the use of low-dose aspirin is now generally recommended for women with chronic hypertension for preeclampsia prevention [28,29].

Finally, long-term metabolic and endocrine follow-up should be considered for both the mother and newborn, including after pregnancy. 

The major strength of this large population-based study is that it enabled a good resolution for every aspect of placenta-mediated complications, given that the large number of patients provides an adequate sample size to reach a clear statistical result that, perhaps, may be generalized for other populations as well. The results of this study represent epidemiological data collected over three decades in the Negev population in Israel, where SUMC serves as the only tertiary hospital. As the largest birth center in Israel, it allows for the investigation of large, non-selective, population-based studies. In Israel, the healthcare system is universal, allowing all residents to utilize medical facilities covered by a national health insurance that is mandatory by law, and hospitalizations are free of charge. This unique setting enabled all women who resided in the area to attend our hospital, which later translated into a heterogenous cohort of women that is based on a non-selective population. Hence, the balanced sample of the population that reside within the area and treated in our institution also contributed to the external validity of the study and strengthened the generalizability of our results. 

In addition, as a single-center study, one can assume that all patients in this study received the same medical considerations by the attending obstetricians and were followed by the same protocols when managing patients with and without chronic hypertension during labor, excluding the possibility that some patients of the cohort were managed using different protocols in other institutions.

One limitation of this study is that the data collected in this research did not distinguish between patients with chronic hypertension who received antihypertensive treatment or those who began their pregnancy with well-controlled hypertension, and those who did not receive antihypertensive treatment or had uncontrolled hypertension at the start of their pregnancy. Despite controversy in the literature about antihypertensive treatment during pregnancy for women who develop non-severe hypertension [30], there is a general consensus about antihypertensive treatment during pregnancy for women with even mild chronic hypertension. An important study recently published in the New England Journal of Medicine (NEJM) showed that antihypertensive treatment for mild chronic hypertension during pregnancy decreased rates of preterm delivery and preeclampsia compared to the control group that was only treated if severe hypertension was developed [31]. Additionally, low-dose aspirin treatment in women at high-risk to develop preeclampsia is also associated with a reduced risk of preeclampsia and the disease’s numerous harms [32,33]. However, as mentioned earlier, the benefit of low-dose aspirin for preeclampsia prevention in the setting of chronic hypertension is still controversial, although there is a general agreement that these high-risk women might benefit from preventive treatment. We acknowledge that in this retrospective study, information regarding the treatment of patients, either antihypertensive medications or low-dose aspirin, was unavailable in our dataset, and further research is needed to test this impact on the development of other placenta-mediated complications. 

According to Bartsch et al. [6], one of the most important risk factors for preeclampsia is having had preeclampsia in previous pregnancies, as in their study, those with prior preeclampsia had the greatest pooled relative risk for preeclampsia in the index pregnancy. Unfortunately, we had no data on prior preeclampsia as this diagnosis was not listed in our database. However, in our multivariable models, we used a GEE method to account for women who had multiple deliveries, so those who had preeclampsia in one of their pregnancies were also later accounted for in their remaining pregnancies. This, in part, adjusts for recurrent occurrences of preeclampsia in pregnancies. 

Another limitation is that our study did not include environmental exposure, lifestyle factors, and socioeconomic status, all of which may play an important role in many health aspects of childbearing age women. These factors are virtually impossible to control in a retrospective study, but since the study population resided in the same area, it was possible to presume some similar exposures, hopefully having only minimal effect on the results.

Of note, this research examined the correlation between chronic hypertension in pregnancy and the development of placenta-mediated complications without consideration of the severity of hypertension. Since the development of maternal complications (that are not placenta-mediated), such as pulmonary edema, cerebrovascular accident, or renal failure, is higher among patients with severe chronic hypertension and not with mild hypertension [16], examining the correlation between the severity of hypertension and the occurrence of placenta-mediated complications warrants further research.

## 5. Conclusions

In our cohort, chronic hypertension in pregnancy was found to be a statistically significant, independent risk factor for placenta-mediated complications, regardless of preeclampsia, as we carefully investigated each of these outcomes adjusting for the new development of preeclampsia in this cohort of high-risk women.

In an era of advanced maternal age, due to delayed childbearing and the increasing use of artificial reproductive techniques, it is expected that we will encounter more pregnant women with pre-existing hypertension that will require our attention for increased surveillance and counseling. Enhancing our understanding of these intricate relationships is pivotal in devising effective strategies aimed at optimizing maternal and fetal outcomes in this high-risk cohort.

## Figures and Tables

**Table 1:** Demographic characteristics of women with and without chronic hypertension.

	Chronic Hypertension (*n* = 3949)	No Chronic Hypertension (*n* = 352,407)	*p* Value
Maternal age (years, ±SD)	32.8 ± 6.2	28.2 ± 5.8	<0.001
Nulliparity, n (%)	677 (17.1)	85,397 (24.2)	<0.001
Obesity, n (%)	172 (4.4)	3905 (1.1)	<0.001
Smoking, n (%)	28 (0.7)	2522 (0.7)	0.961
Pre-gestational diabetes, n (%)	288 (7.3)	3130 (0.9)	<0.001
Fertility treatments, n (%)	229 (5.8)	7482 (2.1)	<0.001

**Table 2:** Pregnancy outcomes of women with and without chronic hypertension.

	Chronic Hypertension (*n* = 3949)	No Chronic Hypertension (*n* = 352,407)	Odds Ratio	95% Confidence Interval	*p* Value
Gestational age at birth (weeks, ±SD)	37.9 ± 2.5	39.0 ± 1.9			<0.001
Preterm delivery (<37 weeks), n (%)	731 (18.5)	23,780 (6.8)	3.14	2.89–3.40	<0.001
Induction of labor, n (%)	1552 (39.3)	74,128 (21.0)	2.43	2.28–2.59	<0.001
Fetal growth restriction (FGR), n (%)	177 (4.5)	6048 (1.7)	2.69	2.30–3.13	<0.001
Birthweight (grams, ±SD)	3091 ± 754	3201 ± 515			<0.001
Low birthweight (<2500 g), n (%)	620 (15.7)	24,056 (6.8)	2.54	2.33–2.77	<0.001
Gestational diabetes, n (%)	653 (16.5)	12,939 (3.7)	5.19	4.77–5.66	<0.001
Preeclampsia, n (%)	852 (21.6)	12,869 (3.7)	7.26	6.71–7.85	<0.001
Severe preeclampsia ^1^, n (%)	399 (10.1)	2776 (0.8)	14.15	12.68–15.80	<0.001
Eclampsia, n (%)	3 (0.1)	101 (0.0)	2.65	0.84–8.36	0.083
Placental abruption, n (%)	45 (1.1)	1811 (0.5)	2.23	1.66–3.00	<0.001
Cesarean delivery, n (%)	1455 (36.8)	48,467 (13.8)	3.66	3.42–3.90	<0.001
Instrumental delivery, n (%)	30 (0.8)	7332 (2.1)	0.36	0.25–0.51	<0.001
Low Apgar score (<7), n (%)	40 (1.0)	1990 (0.6)	1.80	1.32–2.47	<0.001
Postpartum hemorrhage (PPH), n (%)	31 (0.8%)	2046 (0.6%)	1.35	0.94–1.93	0.093
Perinatal mortality, n (%)	60 (1.5)	2764 (0.8)	1.95	1.51–2.52	<0.001

^1^ Defined using the ACOG definition of preeclampsia with severe features [22]: Systolic blood pressure of 160 mm Hg or more, or diastolic blood pressure of 110 mm Hg or more on two occasions at least 4 h apart (unless antihypertensive therapy is initiated before this time). Thrombocytopenia (platelet count less than 100 × 10^9^/L). Impaired liver function that is not accounted for by alternative diagnoses and as indicated by abnormally elevated blood concentrations of liver enzymes (to more than twice the upper limit normal concentrations) or by severe persistent right upper quadrant or epigastric pain unresponsive to medications. Renal insufficiency (serum creatinine concentration of more than 1.1 mg/dL or a doubling of the serum creatinine concentration in the absence of other renal disease). Pulmonary edema. New-onset headache unresponsive to medication and not accounted for by alternative diagnoses. Visual disturbances.

**Table 3:** Results of the GEE models (cluster analysis) for the association between chronic hypertension and placenta-mediated complications, adjusted for preeclampsia and other confounders.

	Crude Odds Ratio	95% Confidence Interval	Adjusted * Odds Ratio	95% Confidence Interval	*p* Value
Preeclampsia **	7.26	6.71–7.85	3.58	3.18–4.03	<0.001
Fetal growth restriction (FGR)	2.69	2.30–3.13	1.59	1.40–1.79	<0.001
Placental abruption	2.23	1.66–3.00	1.26	0.92–1.72	0.144
Preterm delivery	3.14	2.89–3.40	2.29	2.09–2.52	<0.001
Perinatal mortality	1.95	1.51–2.52	1.66	1.25–2.21	<0.001

* Adjusted for preeclampsia, maternal age, smoking status, use of fertility treatment, diabetes mellitus (pre-gestational or gestational), obesity, parity, and year of inclusion to the study (child birth year). ** Adjusted for maternal age, smoking status, use of fertility treatment, diabetes mellitus (pre-gestational or gestational), obesity, parity, and year of inclusion to the study (child birth year).

## Data Availability

The original contributions presented in the study are included in the article/Supplementary Material, further inquiries can be directed to the corresponding author.

## Supplementary Materials

The following supporting information can be downloaded at: https://www.mdpi.com/article/10.3390/jcm13041111/s1, Figure S1. Flow chart for the inclusion and exclusion criteria of the study population; Figure S2. Rates of placental abruption; Figure S3. Rates of fetal growth restriction; Figure S4. Rates of preterm delivery; Figure S5. Rates of preeclampsia; Figure S6. Rates of perinatal mortality.
