# Supplementary material for: Chronic Hypertension in Pregnancy and Placenta-Mediated Complications Regardless of Preeclampsia
Source: J Clin Med. 2024 Feb 16;13(4):1111. doi: 10.3390/jcm13041111 (PMC10889586; doi:10.3390/jcm13041111)
Supplement: Supplementary file 1 [file jcm-13-01111-s001.zip › jcm-2841828-supplementary.pdf]

Journal of
Clinical Medicine

MDPI

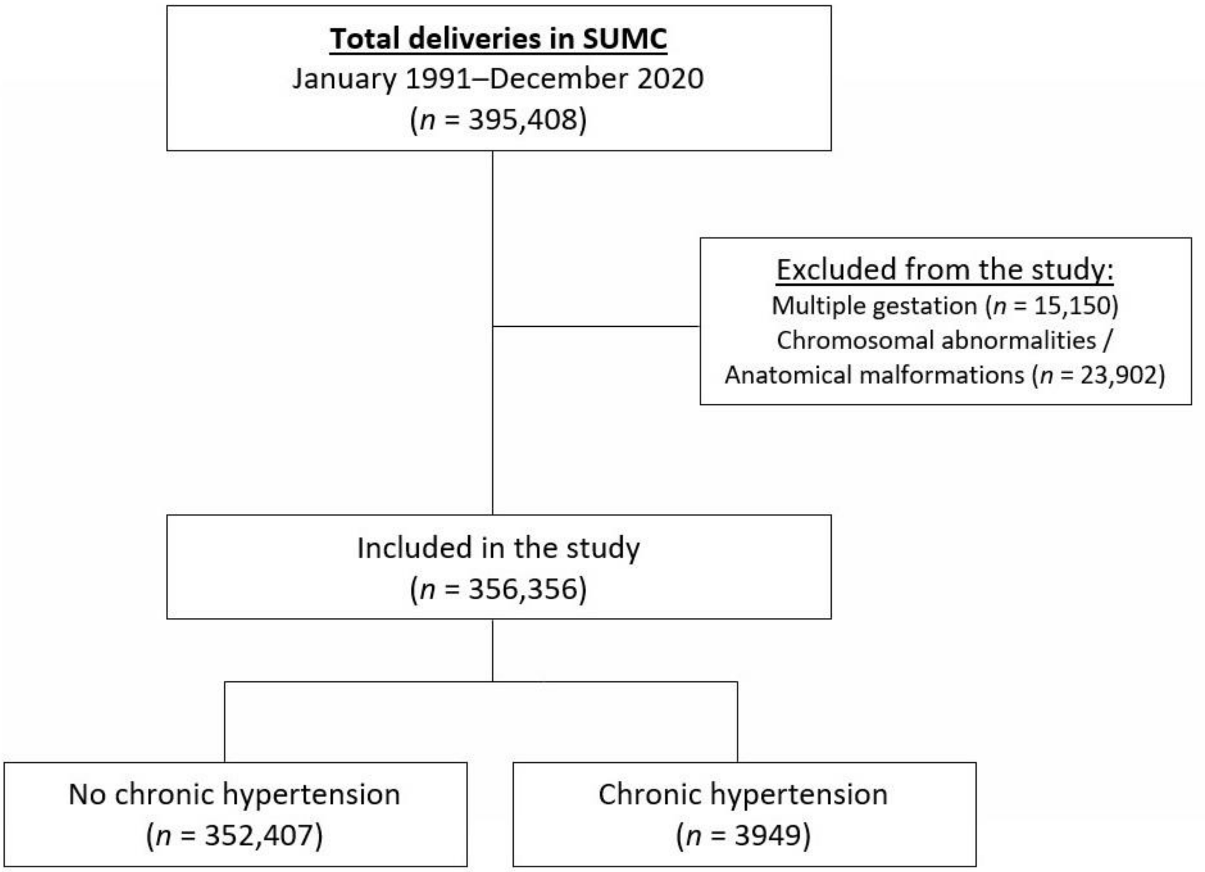

Figure S1. Flow chart for the inclusion and exclusion criteria of the study population.

The following figures represent the annual trends of selected placenta-mediated outcomes investigated in this study:

Figure S2 presents annual rates of placental abruption among the group of women with chronic hypertension. During the study period, an average annual decrease of -1.3% was noted.

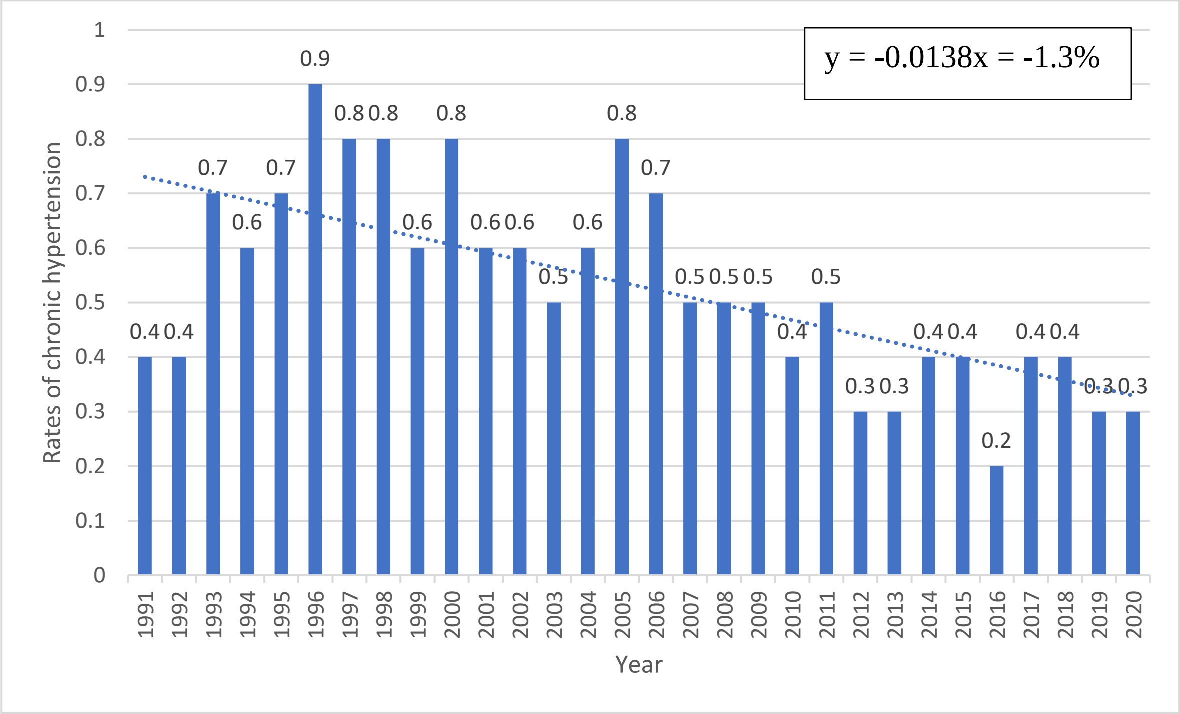

Figure S2. Rates of placental abruption (% of study population per year).

Figure S3 presents annual rates of fetal growth restriction among the group of women with chronic hypertension. During the study period, an average annual decrease of -5.1% was noted.

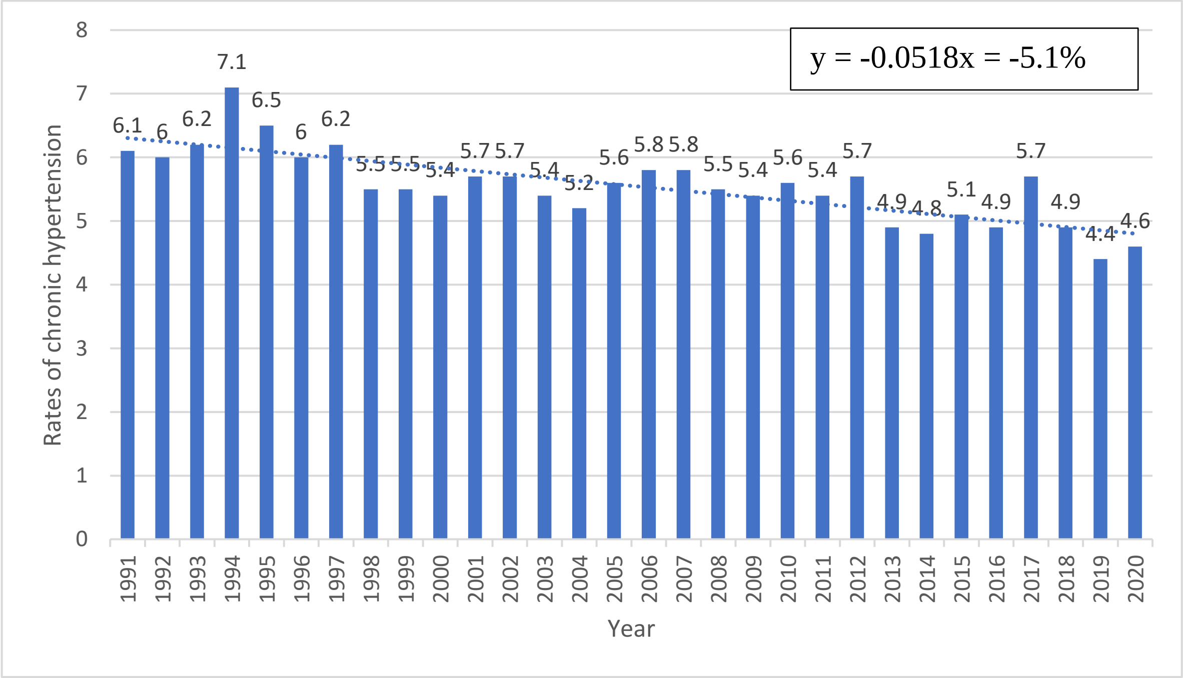

Figure S3. Rates of fetal growth restriction (% of study population per year).

Figure S4 presents annual rates of preterm delivery among the group of women with chronic hypertension. During the study period, an average annual decrease of -0.5% was noted.

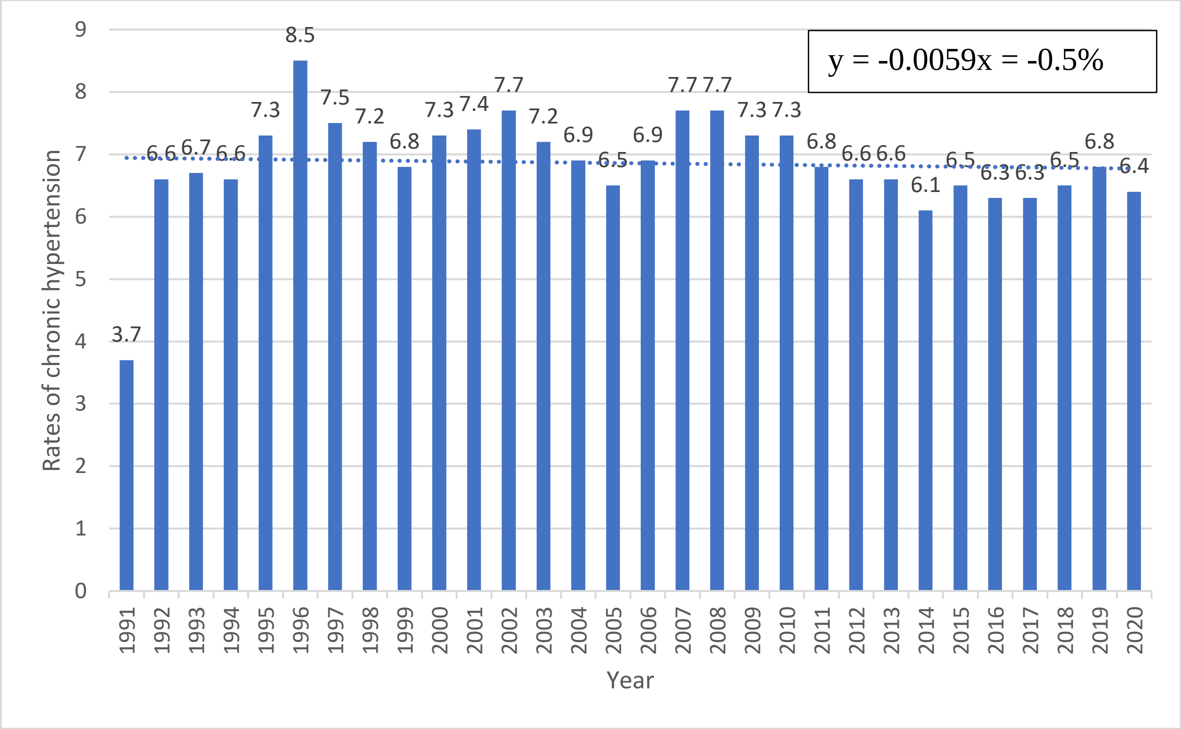

Figure S4. Rates of preterm delivery (% of study population per year).

Figure S5 presents annual rates of preeclampsia among the group of women with chronic hypertension. During the study period, an average annual decrease of -5% was noted.

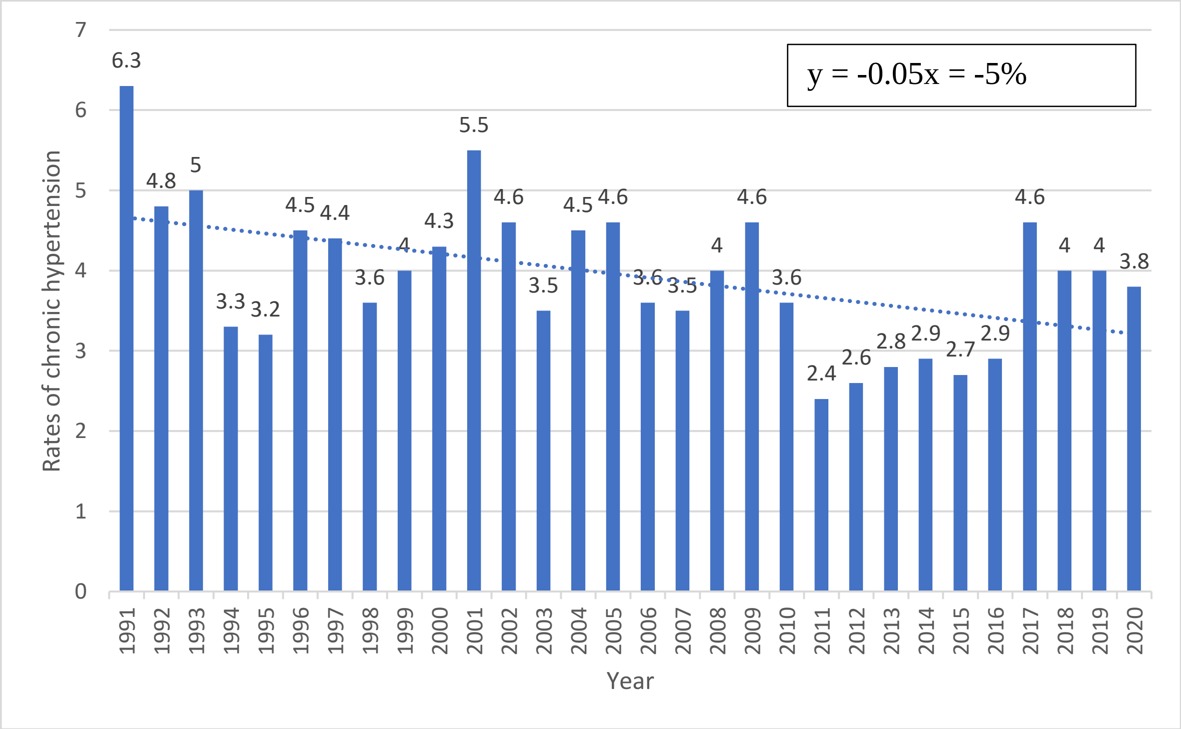

Figure S5. Rates of preeclampsia (% of study population per year).

Figure S6 presents annual rates of perinatal mortality among the group of women with chronic hypertension. During the study period, an average annual decrease of -0.06% was noted.

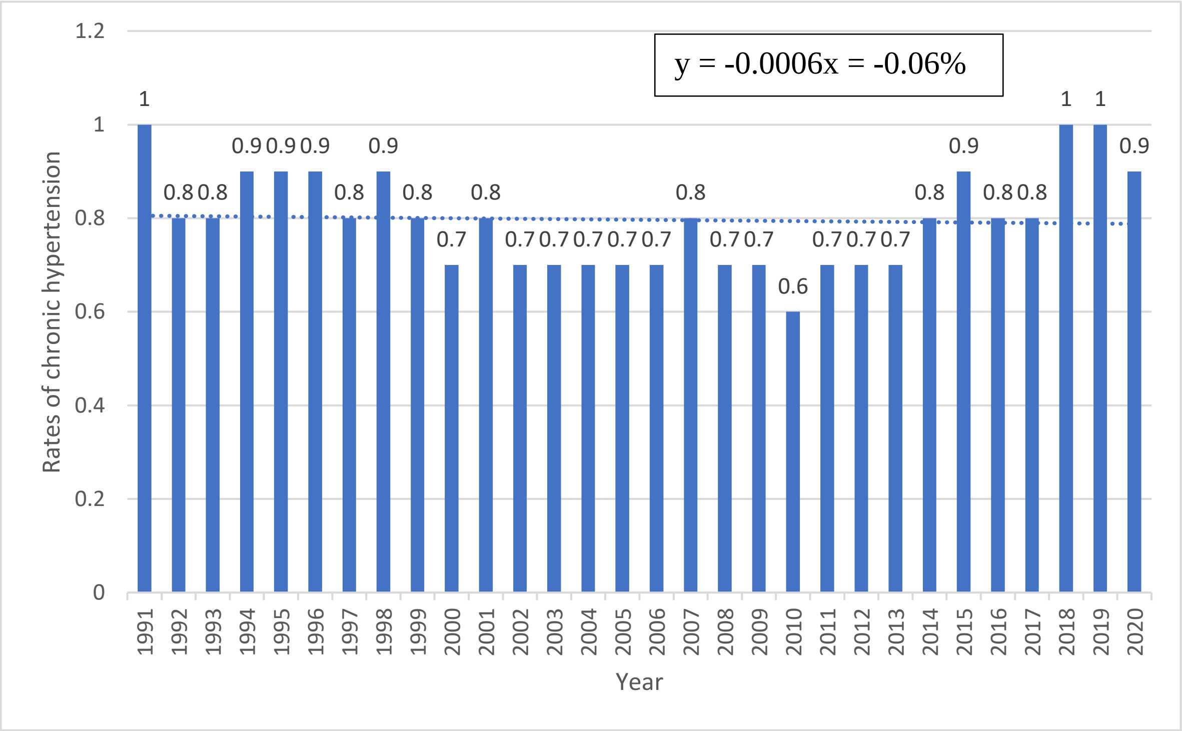

Figure S6. Rates of perinatal mortality (% of study population per year).
